# Mutations in the *LHX2* gene are not a frequent cause of micro/anophthalmia

**Published:** 2010-12-18

**Authors:** Annaïck Desmaison, Adeline Vigouroux, Claudine Rieubland, Christine Peres, Patrick Calvas, Nicolas Chassaing

**Affiliations:** 1 INSERM, U563, Centre de Physiopathologie de Toulouse Purpan, Toulouse, France; 2 CHU Toulouse, Hôpital Purpan, Service de Génétique Médicale, Toulouse, France; 3 Université Toulouse III Paul-Sabatier, Toulouse, France; 4 Division of Medical Genetics, Centre Hospitalier Universitaire Vaudois, Lausanne, Switzerland

## Abstract

**Purpose:**

Microphthalmia and anophthalmia are at the severe end of the spectrum of abnormalities in ocular development. A few genes (orthodenticle homeobox 2 [*OTX2*], retina and anterior neural fold homeobox [*RAX*], SRY-box 2 [*SOX2*], CEH10 homeodomain-containing homolog [*CHX10*], and growth differentiation factor 6 [*GDF6*]) have been implicated mainly in isolated micro/anophthalmia but causative mutations of these genes explain less than a quarter of these developmental defects. The essential role of the LIM homeobox 2 (LHX2) transcription factor in early eye development has recently been documented. We postulated that mutations in this gene could lead to micro/anophthalmia, and thus performed molecular screening of its sequence in patients having micro/anophthalmia.

**Methods:**

Seventy patients having non-syndromic forms of colobomatous microphthalmia (n=25), isolated microphthalmia (n=18), or anophthalmia (n=17), and syndromic forms of micro/anophthalmia (n=10) were included in this study after negative molecular screening for *OTX2, RAX, SOX2*, and *CHX10* mutations. Mutation screening of *LHX2* was performed by direct sequencing of the coding sequences and intron/exon boundaries.

**Results:**

Two heterozygous variants of unknown significance (c.128C>G [p.Pro43Arg]; c.776C>A [p.Pro259Gln]) were identified in *LHX2* among the 70 patients. These variations were not identified in a panel of 100 control patients of mixed origins. The variation c.776C>A (p.Pro259Gln) was considered as non pathogenic by in silico analysis, while the variation c.128C>G (p.Pro43Arg) considered as deleterious by in silico analysis and was inherited from the asymptomatic father.

**Conclusions:**

Mutations in *LHX2* do not represent a frequent cause of micro/anophthalmia.

## Introduction

Microphthalmia and anophthalmia are at the severe end of the spectrum of abnormalities in ocular development. The combined occurrence rate for these two malformations is estimated between 3 and 30 per 100,000 births [1]. Mutations in several genes have been found in syndromic and non-syndromic anophthalmia. Heterozygous mutations in SRY-box 2 (*SOX2*) account for approximately 10% of anophthalmias [2,3]. Growth differentiation factor 6 (*GDF6*) mutations may account for up to 8% of micro/anophthalmia [4,5]. Other genes have been identified as causing isolated anophthalmia or microphthalmia in humans (orthodenticle homeobox 2 [*OTX2*], retina and anterior neural fold homeobox [*RAX*], and CEH10 homeodomain-containing homolog [*CHX10*]) [1,3,6-8]. These latter are implicated in a very small proportion of affected individuals, implying wide genetic heterogeneity to match the phenotypic variability.

The LIM homeobox 2 (LHX2) transcription factor has been shown to be essential for mammalian eye development and mice deficient in functional Lhx2 protein have been shown to display anophthalmia [9]. More recent studies on mouse models have demonstrated that, during eye development, Lhx2 regulates levels and/or expression patterns of Rax, Chx10, Sox2, and Otx2 [10,11], themselves involved in human micro/anophthalmia.

We hypothesize that mutations in *LHX2* could lead to severe eye developmental disorders including micro/anophthalmia. We thus performed molecular analysis in 70 micro/anophthalmia patients for whom previous molecular analysis of genes implicated in isolated micro/anophthalmia (*SOX2*, *OTX2*, *RAX*, and *CHX10*) failed to identify any causative mutation.

## Methods

### Patients

Seventy patients having non-syndromic forms of colobomatous microphthalmia (n=25), isolated microphthalmia (n=18), or anophthalmia (n=17), or syndromic forms of micro/anophthalmia (n=10) were included in this study. Their informed consent was obtained beforehand, according to French law. Micro/anophthalmia was considered as syndromic when the patient presents at least one other non ocular malformation (in our patients, associated malformations were intestinal atresia multiple, corpus callosum agenesis, heart malformation, deafness, Dandy Walker malformation, labiopalatal cleft, sexual ambiguity, hypospadias, arthrogryposis, and choanal atresia). All patients included had undergone molecular analysis of *SOX2*, *OTX2*, *RAX, CHX10*. Direct sequencing of the coding regions and exon/intron boundaries and exclusion of exonic rearrangement by Quantitative Multiplex PCR of Short fluorescent Fragments (QMPSF) of these genes failed to identify any causative mutation in these patients.

### Techniques

The 5 exons of the *LHX2* gene were amplified by PCR using primers deduced from the *LHX2* genomic sequence. Primer pairs and PCR conditions used are summarized in Table 1. Products were amplified in 25 µl reactions containing 50 ng genomic DNA, 1× PCR buffer, 0.2 mM dNTPs, 2 mM MgCl_2_, 100 nM forward primer, 100 nM reverse primer, and 1 U of Taq polymerase. Betaine (1 M) was added in PCR mix for exons 1 and 2. All PCR reactions were performed with a 5 min 95 °C denaturing step, followed by 14 cycles of 95 °C for 30 s, annealing temperature for 30 s (70 °C to 62 °C, −0.5 °C/cycle) and 72 °C for 45 s, followed by 20 cycles of 95 °C for 30 s, 62 °C for 30 s, and 72 °C for 45 s with a final elongation step of 72 °C for 7 min. PCR amplifications were subsequently purified using QIAquick Gel Extraction kit (QIAGEN SA, Courtaboeuf, France), and both forward and reverse strands were sequenced using Big Dye DNA sequencing kit (Applied Biosystems, Warrington, UK). Reactions were analyzed in an ABI3100 sequencer (Applied Biosystems). Sequence variations were numbered with the adenine of the ATG initiation codon as the first nucleotide (the *LHX2* GenBank accession number was NM_004789.3).

**Table 1:** Primers used for *LHX2* molecular analysis.

**Exon**	**Forward primer**	**Reverse primer**	**Product length (bp)**
1	TGAGGCGGGGGGCAAGCCCT	GGAGCCACCGGCCTTGCATT	333
2	ATGTCCTGGCAGCCCCCTCC	GCCAAACTGTAAGACTGTGCCTG	404
3	CCGTGTGTTCCCACAGCCCC	CCGTCGAGGCCGCACACTTT	505
4	TGGGTGGGGCGAGTGTGGAT	GTCCTTCCAAGGCCCACGGC	369
5	CTCACCAGCCCTTCCCTGTC	ATGTGGTTAGTTAGTTGCTC	445

## Results and Discussion

*LHX2* encodes the transcription factor LHX2 which is highly conserved across species [12] and has recently been demonstrated to play a critical role in eye development [10,11]. LHX2 is required to induce or maintain expression of genes required at the early optic vesicle stage for regionalization, establishment of retinal dorsoventral polarity, retinal progenitor cell properties, and lens specification [11]. LHX2 has thus been proposed to link the multiple pathways needed for transition of the optic vesicle to the optic cup [11]. Mice lacking *Lhx2* expression display anophthalmia, and this transcription factor has been involved in regulation of expression levels and/or expression patterns of genes already involved in micro/anophthalmia (*SOX2*, *RAX*, *CHX10*, and *OTX2*) during eye development [10,11]. We hypothesized that mutations in *LHX2* may be involved in human micro/anophthalmia, and thus molecular screening of this gene in 70 micro/anophthalmic patients was performed.

**Figure 1:**
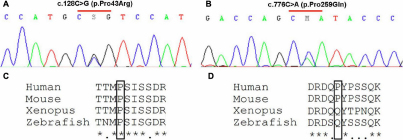
Missense variations identified in this cohort, and conservation of involved amino-acid among species. Electropherograms showing the c.128C>G (p.Pro43Arg; **A**) and the c.776C>A (p.Pro259Gln; **B**) *LHX2* variations. Alignment of part of LHX2 proteins from human, mouse, *Xenopus* and zebrafish, showing conservation of proline 43 (**C**, boxed) in these species, and absence of proline 259 conservation (**D**, boxed), which is replaced by a glutamine in *Xenopus* and zebrafish.

Molecular analysis allowed identification of three sequence variations. We observed presence of the described SNP c.783G>C (p.Pro261Pro) either in a heterozygous or homozygous state in 42 out of 70 patients. In addition, two variants of unknown significance were identified. The heterozygous c.128C>G (p.Pro43Arg; Figure 1A) was identified in an anophthalmic patient originating from Libya for whom no other sequence variation was found. Pro43 amino-acid is conserved among species (Figure 1C) and is located closely to the conserved LIM domain 1 of the protein. This variation was not identified in a panel of 200 control chromosomes of mixed geographical origins (Caucasian, African, and Asian), and was considered as probably damaging by in silico analysis (PolyPhen and SIFT software). However, familial study has shown that this variation was inherited from his father who harbors a normal ocular examination. Thus, this heterozygous variation may be non-pathogenic, even if dominant inheritance with incomplete penetrance can not be totally ruled out. We cannot also exclude presence of an undetected maternally inherited mutation (e.g located in intronic or promoter region sequences, or an exonic rearrangement) fitting with an autosomal recessive inheritance. In a French patient with colobomatous microphthalmia, we identified the heterozygous variation c.776C>A (p.Pro259Gln; Figure 1B). This variation was not identified in a panel of 200 chromosomes from Caucasian controls. No sample was available for his parents, and familial segregation study was not possible. Additionally, there are arguments against the implication of this variation in the patient’s ocular phenotype. First, Pro259 is not a conserved amino-acid among species (Figure 1D), and a glutamine is present at this position in several distant species including *Xenopus*, Chicken, and Fugu Fish [12]. Second, this variation was considered as non damaging by in silico analysis (PolyPhen and SIFT software). Thus, we consider this variant as probably non deleterious. Molecular analysis failed to identify any other sequence variation in the remaining 68 patients included in this study.

In conclusion, although mutations in *LHX2* may nevertheless be implicated in some micro/anophthalmia patients, our results suggest that such sequence variations are not a frequent cause of micro/anophthalmia. Molecular basis of these ocular malformations remains still poorly understood and further work remains to be achieved to identify new micro/anophthalmia genes.
